# Systematization of nursing care: an instrument in the occupational health work process

**DOI:** 10.47626/1679-4435-2021-738

**Published:** 2021-12-30

**Authors:** Karen Cristina Carlos da Silva, Luciana Jerônimo de Almeida Silva, Simone Albino da Silva, Roberta Seron Sanches, Zélia Marilda Rodrigues Resck

**Affiliations:** Enfermagem, Universidade Federal de Alfenas, Alfenas, MG, Brazil.

**Keywords:** worker’s health, occupational health nursing, nursing processes, saúde do trabalhador, enfermagem do trabalho, processos de enfermagem

## Abstract

The environment where workers perform their activities and the way the work is accomplished can harm workers’ health. This study aimed to discuss the systematization of nursing care in occupational health by means of a theoretical, reflective essay on this topic. The discussion was supported by three guiding points: occupational health nursing; the role of occupational health nurses; and the applicability of the systematization of nursing care in occupational health. Occupational health nurses should have adequate technical-scientific knowledge and use the systematization of nursing care as an instrument in their work processes.

## Resumo

O ambiente no qual o trabalhador desenvolve suas atividades e a maneira como o trabalho é realizado podem trazer prejuízos à saúde do trabalhador. Objetivou-se discutir sobre a sistematização da assistência em enfermagem na saúde ocupacional, por meio de um ensaio teórico e reflexivo sobre essa abordagem. Apoiou-se em três pontos norteadores: a enfermagem do trabalho e a saúde ocupacional; a atuação do enfermeiro do trabalho; e a aplicabilidade da sistematização da assistência em enfermagem na saúde ocupacional. É necessário que o enfermeiro do trabalho tenha conhecimento técnico-científico adequado e utilize da sistematização da assistência em enfermagem como um instrumento em seu processo de trabalho.

## INTRODUCTION

In Portuguese, the word ‘work’ has its origin in the Latin word *tripalium*, which is an instrument consisting of three stakes on which farmers beat wheat, but in some dictionaries, we can find this word defined as a torture instrument, as this instrument was later used to torture slaves.^1^

Work can be life changing, as it can interfere positively or negatively with an individual’s life. Working in a healthy work environment can contribute to greater productivity, better quality of service, and workers’ satisfaction. Several factors present in the work environment can contribute to occupational illness, including the lack of collective and personal protective equipment and the lack of knowledge of the occupational risks to which employees are exposed.^2^

In this respect, occupational distress and illness can be related to work relationships, including both the relationship between the individual and the physical environment, in terms of exposure to occupational hazards without proper (collective and personal) protective equipment, and the relationships arising from the work organization (relationships with leaders and colleagues that can cause psychological damage).^1^

The Brazilian Unified Health System (Sistema Único de Saúde, or SUS for short, in Portuguese) advocates that health is the duty of the State and the right of the population, including workers. Ordinance No. 1823 of August 23, 2012, established the National Policy on Occupational Health (Política Nacional de Saúde do Trabalhador e da Trabalhadora, PNSTT), which aims to define the principles, guidelines, and strategies targeted at comprehensive care in occupational health.^3^

The Brazilian Ministry of Labor, through Regulatory Standard 4 (Norma Regulamentadora 4, NR-4), established the Specialized Service in Safety Engineering and Occupational Medicine (Serviço Especializado em Engenharia de Segurança e Medicina do Trabalho, SESMT), which includes a multidisciplinary team consisting of an occupational health nurse, occupational physician, occupational practical nurse, safety engineer, and safety officer, with the purpose of promoting health and protecting the integrity of workers in the workplace.^4^

As for the occupational health nurse, this health professional is known to play a key role in structuring programs and providing occupational health services.^5^

Occupational health is characterized as a field of interdisciplinary practices and strategic knowledge aimed at analyzing and intervening in work relationships that cause illnesses and injuries, with promotion, prevention, and surveillance as a reference point.^6^

In this context, nursing has an important role in the applicability of the systematization of nursing care (SNC) in occupational health, since, as part of the SESMT, these health professionals are responsible for planning and helping to implement actions for occupational health care.

For nurses to play their role properly and safely, they need to have technical-scientific skills and knowledge of the work environment in which employees perform their activities and of the respective health risks associated with this environment.

This theoretical essay is justified on the grounds of the lack of technical-scientific knowledge to apply SNC to occupational health, as it is a mandatory activity for all nurses, whether in the public or private sector, regardless of where the activity is performed, whether in the hospital or occupational health setting, and due to the small number of investigations that address this issue. This discussion may provide knowledge to health professionals, and especially to the nursing staff, thus helping to improve their technical-scientific knowledge of occupational health.

Therefore, the present study aimed to discuss SNC in occupational health by means of a theoretical, reflective essay on this topic.

## METHODS

This is a theoretical, reflective, opinion essay on the implementation of SNC in occupational health. The theoretical essay is based on a logical and reflective presentation, with careful reasoning, in addition to a high level of personal interpretation and criticism.^7^

This study was based on the authors’ evaluations and critical perceptions of the subject, with support from the literature. Given the characteristics of the study, it was exempt from research ethics committee approval.

The reflection on SNC applied to occupational health was supported by three guiding points: occupational health nursing; the role of occupational health nurses; and the applicability of SNC in occupational health.

## OCCUPATIONAL HEALTH NURSING

Since ancient times, there has been a concern to protect people from obstacles that may arise while engaged in professional activities, such as accidents, illness, and even death. These obstacles crossed the centuries and the different civilizations, resulting in laws that seek to place work in a context of norms that allow workers to perform their activities safely.^8^

However, occupational health and safety is not always seen as a priority. Most companies consider only profitability. They find it expensive to send workers out of the company to perform occupational health examinations, as well as the cost of health examinations, in addition to the adjustments that often have to be made in the workplace to make it a healthy environment for work activities.

In order for the work to be performed under conditions of immediate safety and free from illness in the medium and long term, the SESMT must be implemented, which includes, among other health professionals, the occupational health nurse.^9^

The role of occupational health nurses is closely related to the prevention and promotion of occupational health, as well as to protection against the risk of accidents caused by chemical, physical, biological, and psychosocial agents. Nursing care in the work environment is essential, as it significantly contributes to reducing the number of accidents and diseases that can affect workers, thus improving quality of life and work performance.^10^

However, occupational health nurses may encounter obstacles in their professional performance. Therefore, it is important to develop leadership and decision-making skills and to be flexible in conflict situations, taking risks, preparing and innovating to search for new possibilities, negotiating, and even modifying the work environment in order to provide health benefits for all involved in the work process.^10^

It should be noted that, in many situations, nurses and other health professionals who play a role in the occupational setting do not have their importance duly recognized, in addition to facing the need to mediate interests and demands between workers, employers, and labor legislation. The role of the occupational health nurse needs greater recognition due to its importance in decision-making regarding the procedures and approaches that should be adopted in relation to occupational health and safety.^11^

The employer may not invest as much as it would be necessary, which can break the continuity of nurses’ work, leading to insufficient recognition of their work. This lack of recognition results from the trivialization of norms and legislation on safety at the workplace and of their importance for employers, who complain about increased expenditure, and for workers, who resist following the rules.^11^

Therefore, occupational health nurses are faced with the difficulty of making both parties aware of their duties. Resistance to developing a culture of safety can affect work in the workplace.

## THE ROLE OF OCCUPATIONAL HEALTH NURSES

Occupational health is recognized as a field of interdisciplinary, multiprofessional, interinstitutional practices and strategic knowledge (technical, social, political, and human) aimed at analyzing and intervening in work relationships that cause illnesses and injuries.^4^

In Brazil, the legislation that guarantees workers’ rights is the Consolidation of Labor Laws (Consolidação das Leis do Trabalho, CLT), which specifies the rules for the contract agreement between employers and employees. In 1978, the Brazilian Ministry of Labor and Welfare developed the NRs, which establish the minimum conditions for work environments in order to promote occupational safety and health.^12^

The NRs are mandatory for private companies and may generate fines if the rules are not followed. The most widely known standards are NR-7 - Occupational Health Medical Control Program (Programa de Controle Médico de Saúde Ocupacional, PCMSO) and NR-9 - Environmental Risk Prevention Program (Programa de Prevenção de Riscos Ambientais, PPRA).^4^

Nurses, together with occupational physicians, have their training based on risk management, promotion, and rehabilitation of workers’ health.^13^

Currently, most employers see occupational health as costly and unnecessary, being implemented only for legal compliance. Studies have demonstrated irregularities and inconsistencies in documents related to the field of occupational health and safety. This can contribute to an inadequate workplace that favors the development of diseases.^14^

Some aspects of the NRs have been poorly discussed and can lead to misinterpretations, while non-compliance with the law can lead to a large number of ill and injured workers. It is necessary to discuss and analyze these texts for clarification and changes, thus facilitating the application of legislation.^14^

Occupational health nurses have technical-scientific skills to contribute to workplace awareness actions, performance and analysis of occupational health examinations, history taking, physical examination, and interpretation and application of NRs, thus being a transforming agent for improving workers’ quality of life.

## APPLICABILITY OF THE SYSTEMATIZATION OF NURSING CARE IN OCCUPATIONAL HEALTH

The Brazilian Federal Board of Nursing established the SNC through Resolution No. 272/2002, which was revoked by the currently in force Resolution No. 358/2009. This resolution provides for the SNC and implementation of the nursing process in public and private work environments, where professional nursing care occurs, and for other measures.^15^

SNC is a management tool that guides nurses in their care actions in order to offer quality care in their approaches, along with their technical-scientific knowledge.^16^

The nursing process consists of a basic nursing work instrument to be used in any scenario of direct customer care, guided by at least one nursing theory and composed of ordered, sequential, dynamic, interrelated, interdependent steps. It is used to systematize care directed at the individual, family, or community. It is important to note that nursing care can be systematized at any level of care, that is, organized with protocols, rules, and procedures.^17^

Therefore, SNC is considered an advance for nursing as a profession, as it offers nurses autonomy and allows them to be closer to the person receiving care by delivering the required care systematically. It is nurses’ only opportunity to achieve professional autonomy, being the core of their praxis.^18^

SNC has been partially or fully applied by occupational health nurses, thus contributing to the strengthening of the care provided in a systematic manner and of the professional autonomy of nurses within the institutions.^19^

Although SNC is present in nursing care focused on workers’ health, it is believed that occupational health nurses and organizations need to value the instrument and the nursing process. SNC is viewed as a possibility to be incorporated into occupational health services and also as a management and quality tool for health services offered within companies.^16^

When applied correctly, it allows the company to treat each person holistically and to characterize each worker within their different forms of illness, both physically and mentally.

Occupational health nurses can be a transforming agent in the current scenario when they apply the SNC properly by detecting early signs and symptoms of illnesses and identifying risk factors so that the employee’s role can be adjusted to an activity compatible with the worker’s health condition.

Despite its importance and mandatory nature, nurses find it difficult to apply the SNC to the work environment. This results from several factors, including the lack of time due to work overload and lack of knowledge and specific training. In occupational health, nursing records facilitate the early identification of risk factors for workers’ health and allow for affirmative interventions that can contribute to improving the care provided.^16^

The great challenges faced by health professionals working in the field of occupational health include dealing with the lack of autonomy, the lack of sensitivity on the part of employers to intervene in environmental and working conditions, and their non-compliance with legislation that aims to implement public policies targeting occupational health.^20^

## Final considerations

Occupational health nurses have a key role within a company. They participate in administrative/management activities, educational activities, actions related to nursing procedures, as well as in the promotion, protection, and recovery of workers’ health, spending most of their time in management tasks, followed by consulting activities.

SNC directly contributes to the valuation and autonomy of occupational health nurses within an institution. It allows for the development of health actions and contributes to the continuity of care by a multidisciplinary team. It strengthens workers’ daily care, improving their quality of life and the implementation of public policies.

This allows for an improvement in the quality of the services provided by occupational health nurses and, consequently, for the successful implementation of SNC within companies.
